# Pre-Pandemic- and During-COVID-19 Anxiety and Depression Symptoms and Health-Related Quality of Life Were Similar in Injured Older Adults Enrolled in a Clinical Trial

**DOI:** 10.3390/geriatrics11040090

**Published:** 2026-07-18

**Authors:** Damaris Ortiz, Anthony J. Perkins, Sujuan Gao, Nicholas Adam Sergiwa, Emma Holler, Ashley D. Meagher, Sanjay Mohanty, Dustin D. French, Sue Lasiter, Babar Khan, Malaz Boustani, Ben Zarzaur

**Affiliations:** 1 Department of Surgery, Indiana University School of Medicine, 545 Barnhill Dr., Emerson Hall, Indianapolis, IN 46202, USA; nsergiwa@iu.edu (N.A.S.); mohantys@iu.edu (S.M.); 2 Surgical Outcomes and Quality Improvement Center, Department of Surgery, Indiana University School of Medicine, 545 Barnhill Dr., Emerson Hall, Indianapolis, IN 46202, USA; 3 Department of Biostatistics and Health Data Science, Indiana University School of Medicine, 410 West 10th Street, Suite 3000, Indianapolis, IN 46202, USA; antperki@iu.edu (A.J.P.); sgao@iu.edu (S.G.); 4 Indiana University Health, Methodist Hospital Level One Trauma Center, 1701 Senate Ave., Indianapolis, IN 46202, USA; 5 Department of Ophthalmology, Feinberg School of Medicine, Northwestern University, Chicago, IL 60611, USA; dustin.french@northwestern.edu; 6 School of Nursing and Health Studies, Health Sciences District, University of Missouri, Kansas City, MO 64108, USA; lasiterr@umkc.edu; 7 Department of Medicine, Indiana University School of Medicine, 1120 W. Michigan St., CL 260, Indianapolis, IN 46202, USAmboustan@iu.edu (M.B.); 8 Department of Surgery, University of Wisconsin School of Medicine, Madison, WI 53726, USA; zarzaur@surgery.wisc.edu

**Keywords:** older adults, trauma, depression, anxiety, COVID, quality of life

## Abstract

**Introduction:** The Trauma Medical Home (TMH) randomized clinical trial tested the efficacy of a collaborative care intervention on the biopsychosocial recovery of older injured adults compared to usual care. Study enrollment occurred between 11 October 2017 and 13 October 2021 and study operations were impacted by the COVID-19 pandemic. It is important to measure the potential effects of COVID-19 on research study participants to ensure accurate reporting of results. The purpose of this secondary analysis was to compare depression and anxiety symptoms and health-related quality of life in older adults at TMH study enrollment pre-COVID-19 and during COVID-19. We hypothesized that those enrolled during COVID-19 would have worse symptoms and quality of life. **Methods:** This study was a secondary analysis of data from a multicenter randomized clinical trial (R01AG052493-01A1) TMH, that examined the ability of a collaborative care model to enhance the recovery of older injured adults compared to usual care. It was hypothesized that the TMH intervention would improve biopsychosocial recovery for injured older adults compared to usual care. The enrollment periods pre-COVID-19 and during COVID-19 were compared. Chi-square tests and Wilcoxon Rank Sum tests, then multivariable linear and logistic regressions were completed with SF-36 component scores and depression (PHQ-9) and anxiety (GAD-7) scores as the dependent variables, respectively. **Results:** A total of 429 participants were analyzed. There was no significant difference in anxiety and depression symptoms between the pre-COVID-19 and during-COVID-19 enrollment periods. Perceived limitations due to physical health problems were worse for those enrolled during COVID-19 (adjusted β = −9.8, 95% CI −18.3 to −1.4), but other health-related quality of life measures were similar between the two groups. **Conclusions:** These findings support the conclusion that the negative psychological impacts of the pandemic are likely due to individual factors other than age or COVID-19. These results suggest a high degree of psychological resiliency in older adults under stress. Future research should focus on the potential effects of socioeconomic status, demographic background, and level of physical functioning on psychological outcomes.

## 1. Introduction

The COVID-19 pandemic presented unique challenges to older adults, including increased risk of social isolation, reduced physical activity, and heightened anxiety and depression due to their increased vulnerability to infection [1,2,3]. Research on the impact of COVID-19 on mental health outcomes in older adults is limited and has shown varying results. According to data from the Irish Longitudinal Study on Ageing, depression symptoms in adults more than doubled from a baseline of 7.2% to 19.8% during the COVID-19 pandemic [4]. In a survey of 515 U.S. adults between 20 March and 19 April 2020, older adults had more COVID-19-related stress due to concerns about contracting the virus compared to younger adults [5]. However, older adults also reported more pro-active coping than the younger adults [5]. Another study demonstrated a significant decline in physical and cognitive function among older adults who were hospitalized with COVID-19 and survived [6]. Furthermore, few studies have compared mental health outcomes and health-related quality of life in older adults before and during the pandemic and have found mixed results [7,8]. A secondary analysis of a clinical trial studying outcomes of caregivers of older adults with dementia enrolled participants before and during COVID-19 and found no difference in depression or anxiety symptoms between enrollment periods [7]. A study from Hong Kong examined changes in loneliness, depression, anxiety, and insomnia symptoms before and during the pandemic in adult primary care patients at least 60 years old with at least two medical comorbidities [8]. Out of 583 participants who completed pre-COVID-19 and during-COVID-19 assessments, loneliness, insomnia, and anxiety symptoms increased in the during COVID-19 period, while depression symptoms stayed the same [8].

The pandemic disrupted human subjects research and necessitated modifications to study procedures [9,10,11]. Understanding the relationships between a stressor like the COVID-19 pandemic and psychosocial symptoms in older adults during a clinical trial is critical to ensure accurate reporting of trial results. In addition, this study adds to the limited literature about clinical trials conducted during the COVID-19 time period and can provide insight about the older adult population for future research [12]. The purpose of this study was to compare depression and anxiety symptoms and health-related quality of life in older adults at the time of enrollment pre-COVID-19 and during COVID-19. The authors hypothesized that participants enrolled during COVID-19 would have worse depression and anxiety symptoms and health-related quality of life scores compared to those enrolled pre-COVID-19.

## 2. Materials and Methods

### 2.1. Study Population

This was a post hoc analysis of data from Trauma Medical Home (TMH), a multicenter randomized clinical trial (R01AG052493-01A1) that examined the ability of a collaborative care model to enhance the recovery of older injured adults compared to usual care. The main study results have been published and found no difference in the primary outcomes between the intervention and control groups [11]. Patients were enrolled from 11 October 2017 to 13 October 2021 from four level-1 trauma centers in the Midwest United States (Eskenazi Health Hospital, Indiana University Methodist Hospital, Ascension St. Vincent, and University of Wisconsin). Patients were recruited in person at all hospitals except for the period between 20 March and 1 July 2020, when recruitment was completed remotely by telephone. This study was approved by the Indiana University Institutional Review Board (Protocol Version 6: Study # 1612690852; approved 12 April 2019), and informed consent for this secondary analysis was waived. The study was conducted with ethical best practices as described by the Declaration of Helsinki.

### 2.2. Trauma Medical Home Clinical Trial

The goal of collaborative care is to address biopsychosocial needs with evidence-based performance measures to guide targeted intensification of treatment. The Collaborative Care Model was initially developed for the primary care setting to treat depression and comorbid physical health problems [13,14]. This model has been successfully adapted for other patient populations and mental illnesses [15,16]. In older adults, the Collaborative Care Model has been adapted for patients with dementia and other cognitive disorders [17].

The detailed study protocol has been previously published [18]. In brief, this clinical trial included injured adults aged 50 years and older with an injury severity score (ISS) of 9 or greater who resided within 50 miles of the admitting trauma center and had telephone access. The ISS is an anatomically based score ranging from 0 to 75 that summarizes injury severity across body regions, with higher scores indicating more severe injury; an ISS of 9 or greater is generally consistent with at least moderate, often multi-system, injury [19]. There was no upper ISS limit for inclusion, and the highest observed score in this cohort was 38. For the first 19 months of enrollment the ISS threshold was greater than 9; it was then lowered to 9 or greater because of low enrollment and the exclusion of many otherwise eligible patients, which improved recruitment. Patients with significant head injury, neurodegenerative disease, stroke, spinal cord injury, burns, pregnancy, advanced malignancy, alcohol or drug use disorder within the prior 6 months, or sensory impairment that precluded study participation were excluded. Research assistants screened patients daily for inclusion and exclusion criteria at each site, using manual chart review and screening visits with potential participants to identify exclusions, and any questions of eligibility were adjudicated by the site principal investigators. Informed consent was obtained from all participants or their surrogate-decision-makers prior to study enrollment. Enrollment occurred at the time of hospital discharge. Participants were then randomized to either usual care or the TMH intervention.

### 2.3. Exposure and Outcome Variables

The exposure variable of interest was the timing of enrollment, which was categorized as either pre-COVID-19 (prior to 16 March 2020) or during COVID-19 (on or after 16 March 2020). March 16th, 2020, marked the issuance of an executive order by the governor of Indiana to follow the Centers for Disease Control and Prevention (CDC) guidelines regarding the COVID-19 pandemic.

At the time of enrollment, all participants completed baseline assessments which included depression screening by the Patient Health Questionnaire-9 (PHQ-9), anxiety screening by the Generalized Anxiety Disorder Scale (GAD-7), and health-related quality of life assessment by The Short-Form Health Survey (SF-36). The primary outcomes were differences in the presence of mild to severe anxiety (GAD-7 score of 5 or greater), mild to severe depression (PHQ-9 score of 5 or greater) symptoms, and health-related quality of life based on SF-36 component scores at the time of hospital discharge [20]. The PHQ-9 is a nine-item depression scale with a total score from 0 to 27, and the GAD-7 is a seven-item anxiety scale with a total score from 0 to 21. Scores of 5, 10, and 15 represent the cut-off points for mild, moderate, and severe symptoms, respectively, for both scales. Both are derived from the Patient Health Questionnaire and have good internal consistency and validity for the diagnosis of major depression and generalized anxiety disorder [21,22,23,24]. The SF-36 evaluates physical and social functioning, physical and emotional limitations, general and mental health. Each item is scored from 0 to 100 with higher scores indicating better functioning. Items in the same scale are then averaged together to create the 8 individual scale scores: physical functioning, bodily pain, role limitations due to physical health problems, role limitations due to emotional problems, emotional wellbeing, social functioning, energy/fatigue, and general health perceptions. The Physical Composite Score (PCS) and Mental Composite Score (MCS) are scaled to have a mean of 50 [25,26,27]. A single anchor question indicates the perceived change in health [25].

### 2.4. Data Collection

Sex and insurance type were collected from the electronic medical record (EMR) at the time of enrollment. Race, income, education, and marital status were obtained from a patient questionnaire. Injury characteristics, including injury mechanism and Injury Severity Score (ISS), were obtained from chart review and ISS calculated based on the injuries listed in the problem list. Comorbidity burden was represented by the Charlson Comorbidity score, which was obtained using information from the EMR. The Charlson Comorbidity score was modeled as a continuous variable. Pre-existing anxiety and depression were determined retrospectively by review of prior ICD-9 or ICD-10 diagnosis codes and medication history in the EMR. The limited data study set was provided in Supplemental Materials.

### 2.5. Analysis

Chi-square tests for categorical variables and Wilcoxon Rank Sum tests or two-sample t-tests for continuous data were used to compare whether patient characteristics and outcomes differed by enrollment period (pre-COVID-19 vs. during COVID-19). We then performed multivariable linear regressions with the SF-36 component scores as dependent variables (continuous) and multivariable logistic regressions with depression and anxiety as the dependent variables (binary). All models controlled for age, sex, race, marital status, Charlson Comorbidity score, ISS, mechanism of injury, prior depression or anxiety, and COVID-19 time period. A *p*-value of <0.05 was considered statistically significant, and all analyses were performed using SAS 9.4 (Cary, NC, USA). Regression models were estimated using complete cases for the included covariates. Income and education were not included in the regression models, as neither differed significantly between the enrollment periods and the proportion of participants who declined to report income was similar in the two groups.

#### Generative AI Disclosure

No generative AI was used in the study design, data collection, data analysis, or in the interpretation of results.

## 3. Results

### 3.1. Demographics and Clinical Characteristics

Patient characteristics by enrollment period (pre-COVID-19 and during COVID-19) are displayed in Table 1. Most patients were between the ages of 65–79 years old. Patients enrolled pre-COVID-19 were younger than those enrolled during COVID-19. Pre-COVID-19 patients were also less likely to have pre-existing depression or anxiety (based on diagnosis codes and medication review) than patients enrolled during COVID-19 [n = 133 (74%) without depression/anxiety] vs. n = 132 (59%) without depression/anxiety]. Patients enrolled during COVID-19 were more likely to have a fall as the mechanism of injury [n = 146 (65%) vs. n = 97 (48%)] and have a lower ISS [9 (9–13) vs. 12 (9–14)]. The lower ISS score was confounded by the study lowering the ISS score requirement midway through the study.

### 3.2. Primary Outcomes

The SF-36 Role-physical was lower [32.4 (41.2) vs. 41.1 (42.5)] while Role-emotional was higher [71.1 (40.1) vs. 62.7 (41.0)] for patients enrolled during COVID-19. Regardless of time period, the measures of physical functioning were lower than the general population [26,27]. These results are displayed in Table 2.

### 3.3. Multivariable Regression

The regression models adjusted for age, sex, race, marital status, Charlson comorbidity score, ISS, mechanism of injury, prior depression or anxiety, and COVID-19 time period. After adjustment, Role-physical was still significant while Role-emotional lost significance. Female sex was associated with anxiety symptoms and the physical composite score while a prior history of co-occurring depression and anxiety was significantly associated with the mental health composite score and depression and anxiety symptoms. Charlson was associated with most of the outcomes except for pain and physical functioning. These results are displayed in Table 3 and Table 4.

## 4. Discussion

This secondary analysis aimed to compare depression and anxiety symptoms and health-related quality of life of non-neurologically injured older adults enrolled in a randomized clinical trial before and during the COVID-19 pandemic. The findings showed no significant difference in anxiety and depression symptoms between the two enrollment periods. Perceived limitations due to physical health problems were worse for those enrolled during COVID-19.

These findings suggest that negative psychological impacts of the pandemic were likely due to individual factors other than age or COVID-19 itself [28]. Factors that have been associated with increased emotional distress during COVID-19 include pre-existing dual diagnosis of PTSD and depression [29], living alone [4,29], and financial insecurity [30]. While Seibert et al. found no difference between pre-COVID-19 and during-COVID-19 depression and anxiety symptoms in participants in the Caregiver Outcomes of Alzheimer’s Disease Screening (COADS) study, they found that those who felt more financially secure and those with postgraduate degrees had lower depression and anxiety compared to those who could not make ends meet and those with a high school education or less [7]. In contrast, Taylor et al. found no significant difference in psychological resilience in older adults between 2016 and 2020 and found that financial hardship eroded psychological resilience more in White American participants compared to Black American participants [31]. The current study did not have a diverse cohort to reliably examine demographic differences. These findings highlight the importance of considering socioeconomic and demographic differences in clinical trial design and data analysis.

The current study results contradicted the hypothesis that those enrolled during COVID-19 would have worse psychological symptoms, and are consistent with some literature, such as the previously described COADS study [7]. One systematic review showed older adults had lower stress and less negative emotions than younger adults during quarantine, but did not compare between the pre-COVID-19 and during-COVID-19 time periods [32]. Older adults with hearing loss in another study were frustrated by challenges posed by facial masks during the pandemic but found the use of technology to be helpful to mitigate these difficulties [33]. Social technology was also useful for caregivers of partners with dementia during the pandemic [34]. A qualitative pilot study in Germany examined factors related to use of social technology in older adults with dementia and their caregiver dyads. Familiarity with adequate tech support, and the desire to use the technology, were facilitators to using I-CARE, a tablet-based social interaction intervention designed for people with dementia [34]. The current study switched to telephone contact and assessments after 16 March 2020, but did not use other social platforms. The authors do not know how many participants had access to or utilized other social technology, a factor which may have contributed to decreasing depression and anxiety symptoms by mitigating social isolation.

In the current study, the majority of the health-related quality of life measures were similar between the two groups, and perceived emotional health was actually higher for those enrolled during COVID-19. These findings also refuted our hypothesis. Measures of physical functioning were low regardless of the time of enrollment, but perceived role limitations due to physical health were worse for those enrolled during COVID-19. This may reflect participants who had a COVID-19 infection prior to enrollment, and its negative impact on their functional capacity [35]. Alternatively, the emotional distress of the pandemic may have contributed to perceived physical health limitations, particularly for those with multiple comorbidities [8,20]. Charlson comorbidity scores were significantly associated with the SF-36 mental component score more than the physical component score. Older patients with COVID-19 illness in the UK who required mechanical ventilation and had greater social deprivation were at higher risk for physical frailty and reduction in health-related quality of life [36]. In the current study there was no difference in perceived social functioning between those enrolled pre-COVID-19 and during COVID-19 at the time of their baseline assessment. It is possible that TMH participants had strong social networks.

This was a secondary, exploratory analysis of trial data, and multiple outcomes were compared across the two enrollment periods. With the exception of SF-36 role limitations due to physical problems, all comparisons were non-significant (all *p* > 0.05); a formal correction for multiple comparisons would therefore have made these results even less likely to reach significance and would not have altered the interpretation or conclusions of the study.

This study has important implications for future studies of health-related quality of life and psychological symptoms in older adults. The COVID-19 time period was not associated with significant differences in all outcomes as expected. Consistent with other studies, this suggests a degree of psychological resiliency of older adults in hardship [37]. For example, in a longitudinal Canadian study of older adults with at least two medical comorbidities, psychological resilience was associated with less perceived negative impact of the COVID-19 pandemic than social or functional resilience at baseline and throughout the study period [37]. Prior research has found that pre-existing psychiatric and medical comorbidities are strongly associated with negative psychological symptoms during new stressors [8,29]. This study added to the body of literature demonstrating the significant interplay between multiple medical comorbidities and psychological symptoms. Further investigation is needed to understand the potential impact of socioeconomic status, demographic background, and levels of physical functioning on psychological outcomes in older injured adults over time.

This study had some limitations. While this was a multicenter trial, all centers were in the Midwest region of the United States, which may limit generalizability. The study also enrolled a homogeneous population (88.8% non-Hispanic white) which also limits the generalizability of the findings. Selection bias may have affected our results, as over 50% of our participants were married, with no significant difference in marital status between the groups, and most had an annual income of >$25,000 and some form of government or private insurance. The authors did not consider whether those with prior depression and anxiety were receiving any treatment including antidepressants or anxiolytics, which could decrease symptoms in those individuals. The study did not collect data on pre-existing PTSD diagnoses, or on which participants had a COVID-19 infection prior to study enrollment. These are two unmeasured variables that could potentially be associated with anxiety and depression symptoms or quality of life, although they would be unlikely to alter the results given PTSD co-occurrence with anxiety; also, for participants with prior COVID-19 infection they would presumably have recovered prior to enrollment.

## 5. Conclusions

This secondary analysis of data from a randomized clinical trial showed that non-neurologically injured older adults enrolled during the COVID-19 pandemic had similar depression and anxiety symptoms compared to those enrolled prior to COVID-19. Those enrolled during COVID-19 had greater perceived loss of role functions due to physical health. Future studies are needed to evaluate health-related quality of life and psychological symptoms in older adults over time, and to design clinical trials that can accommodate those with different socioeconomic status, demographic backgrounds, and levels of physical functioning.

## Figures and Tables

**Table 1:** Comparisons of demographic and medical history by enrollment period (pre- vs. during COVID-19).

	Overall (n = 429)	Pre-COVID-19 (n = 203)	During COVID-19 (n = 226)	*p*-Value
Age				0.024
50–64	159 (37.1)	86 (42.4)	73 (32.3)	
65–79	192 (44.8)	77 (37.9)	115 (50.9)	
80+	78 (18.2)	40 (19.7)	38 (16.8)	
Sex				0.863
Female	228 (53.1)	107 (52.7)	121 (53.5)	
Male	201 (46.9)	96 (47.3)	105 (46.5)	
Race				0.305
African-American	37 (8.6)	22 (10.8)	15 (6.7)	
Other	4 (0.9)	2 (1.0)	2 (0.9)	
White	387 (90.4)	179 (88.2)	208 (92.4)	
Missing	1	0	1	
Hispanic	3 (0.7)	2 (1.0)	1 (0.4)	0.495
Insurance Type				0.087
Private	119 (27.7)	57 (28.1)	62 (27.4)	
Medicare	115 (26.8)	59 (29.1)	56 (24.8)	
Medicaid	8 (1.9)	6 (3.0)	2 (0.9)	
Medicare + Medicaid	17 (4.0)	11 (5.4)	6 (2.7)	
Medicare + Private	133 (31.0)	56 (27.6)	77 (34.1)	
None	12 (2.8)	7 (3.4)	5 (2.2)	
Other (+gvt)	25 (5.8)	7 (3.4)	18 (8.0)	
Education				0.322
High school equiv or less	139 (33.5)	73 (37.8)	66 (29.7)	
Some college	70 (16.9)	28 (14.5)	42 (18.9)	
Associate, Bachelor, or vocation	130 (31.3)	58 (30.1)	72 (32.4)	
Master or Doctorate	76 (18.3)	34 (17.6)	42 (18.9)	
Missing	14	10	4	
Income				0.665
<$25,000	71 (16.8)	35 (17.2)	36 (16.3)	
$25,000 to <$50,000	76 (17.9)	37 (18.2)	39 (17.6)	
$50,000 to <$75,000	41 (9.7)	15 (7.4)	26 (11.8)	
$75,000 or more	75 (17.7)	36 (17.7)	39 (17.6)	
Don’t know/Not sure	161 (38.0)	81 (39.4)	81 (36.6)	
Missing	5	0	5	
Marital Status				0.152
Married	239 (55.8)	106 (52.2)	133 (59.1)	
Divorced/widow/separated/Single	189 (44.2)	97 (47.8)	92 (40.9)	
Missing	1	0	1	
Injury Characteristics				<0.001
Fall	243 (56.6)	97 (47.8)	146 (64.6)	
Motor Vehicle Accident	132 (30.8)	84 (41.4)	48 (21.2)	
Other	54 (12.6)	22 (10.8)	32 (14.2)	
Prior Depression/Anxiety				0.015
None	265 (65.3)	133 (73.5)	132 (58.7)	
Depression Only	67 (16.5)	25 (13.8)	42 (18.7)	
Anxiety Only	19 (4.7)	5 (2.8)	14 (6.2)	
Depression & Anxiety	55 (13.5)	18 (9.9)	37 (16.4)	
Missing *	23	22	1	
Median Injury Severity Score (IQR)	10 (9–14)	12 (9–14)	9 (9–13)	<0.001
Median Charlson (Range)	0 (0–10)	0 (0–7)	0 (0–10)	0.729

* Prior depression and anxiety were not collected at the start of the study and were added later; because of withdrawals of consent, this variable was not available for all patients.

**Table 2:** Outcomes of interest by COVID-19 timing (n = 429).

	Overall (n = 429)	Pre-COVID-19 (n = 203)	During COVID-19 (n = 226)	*p*-Value
Any Depression, n (%)	211 (49.4)	104 (51.5)	107 (47.6)	0.417
Any Anxiety, n (%)	173 (40.5)	80 (39.6)	93 (41.3)	0.716
SF-36, mean (SD)				
Physical functioning	32.6 (34.1)	31.5 (34.0)	33.6 (34.2)	0.530
Role-physical	36.5 (42.0)	41.1 (42.5)	32.4 (41.2)	0.032
Pain	43.3 (34.6)	45.5 (35.8)	41.2 (33.3)	0.196
General Health	61.7 (22.3)	60.3 (22.8)	63.0 (21.8)	0.217
Vitality	50.5 (23.4)	50.8 (24.7)	50.3 (22.2)	0.830
Social Functioning	62.1 (32.8)	63.1 (32.8)	61.1 (32.7)	0.529
Role-emotional	67.1 (40.7)	62.7 (41.0)	71.1 (40.1)	0.033
Emotional Wellbeing	73.6 (20.5)	74.8 (20.6)	72.6 (20.4)	0.279
PCS	31.0 (12.3)	31.5 (12.3)	30.5 (12.2)	0.408
MCS	52.3 (11.9)	52.0 (12.2)	52.5 (11.5)	0.663

Notes. SF-36 = Short-Form 36 Health survey; PCS = Physical Component Summary; MCS = Mental Component Summary.

**Table 3:** Regression results for SF-36 scales, adjusting for demographic and clinical characteristics (n = 404).

	**Physical Functioning**		**Role-Physical**		**Pain**		**General Health**		**Vitality**	
	**Estimate (95% CI)**	***p*** **-Value**	**Estimate (95% CI)**	***p*** **-Value**	**Estimate (95% CI)**	***p*** **-Value**	**Estimate (95% CI)**	***p*** **-Value**	**Estimate (95% CI)**	***p*** **-Value**
Age										
50–64 (ref)	0.0		0.0		0.0		0.0		0.0	
65–79	−1.9 (−9.8, 6.0)	0.637	−8.2 (−17.8, 1.3)	0.092	−6.2 (−14.2, 1.8)	0.129	0.9 (−3.8, 5.6)	0.709	−2.1 (−7.3, 3.1)	0.424
80+	3.6 (−6.8, 14.1)	0.493	3.2 (−9.5, 15.8)	0.622	8.5 (−2.1, 19.1)	0.115	2.7 (−3.5, 8.9)	0.398	−3.0 (−9.9, 3.8)	0.383
Female	−11.2 (−18.7, −3.8)	0.003	−10.1 (−19.1, −1.1)	0.028	−6.4 (−13.9, 1.1)	0.096	−1.6 (−6.0, 2.9)	0.489	−1.7 (−6.6, 3.2)	0.492
Race										
Black	−0.9 (−13.4, 11.7)	0.892	−1.6 (−16.8, 13.6)	0.837	−1.3 (−14.0, 11.4)	0.841	−10.3 (−17.8, −2.8)	0.007	−3.7 (−11.9, 4.5)	0.379
Other	20.6 (−13.1, 54.4)	0.230	9.8 (−31.1, 50.6)	0.639	18.1 (−16.1, 52.3)	0.299	7.0 (−13.0, 27.1)	0.491	−1.1 (−23.2, 21.0)	0.922
White (ref)	0.0		0.0		0.0		0.0		0.0	
Married	3.9 (−3.2, 11.1)	0.278	0.6 (−8.0, 9.3)	0.885	0.2 (−7.0, 7.5)	0.949	−2.6 (−6.8, 1.6)	0.224	0.03 (−4.6, 4.7)	0.989
Prior anxiety, depression										
None (ref)	0.0		0.0		0.0		0.0		0.0	
Depression only	7.0 (−2.5, 16.5)	0.150	−7.4 (−18.9, 8.5)	0.207	0.5 (−9.1, 10.2)	0.913	−8.9 (−14.6, −3.3)	0.002	−8.8 (−15.0, −2.5)	0.006
Anxiety Only	14.3 (−1.7, 30.4)	0.080	4.5 (−15.0, 23.9)	0.651	1.2 (−15.0, 17.5)	0.881	2.6 (−6.9, 12.1)	0.592	−1.8 (−12.3, 8.8)	0.742
Both	4.6 (−5.8, 15.0)	0.386	−7.5 (−20.1, 5.1)	0.245	−1.5 (−12.1, 9.0)	0.775	−12.0 (−18.2, −5.8)	<0.001	−11.2 (−18.1, −4.3)	0.002
Injury characteristics										
Fall	−6.9 (−18.0, 4.3)	0.227	−5.0 (−18.5, 8.5)	0.470	−4.4 (−15.7, 6.9)	0.444	−4.6 (−11.2, 2.1)	0.176	−6.1 (−13.4, 1.2)	0.103
Motor vehicle accident	−11.7 (−23.3, −0.1)	0.048	−7.5 (−21.5, 6.6)	0.297	−7.7 (−19.5, 4.0)	0.197	−7.7 (−14.5, −0.8)	0.029	−7.7 (−15.2, −0.1)	0.048
Other (ref)	0.0		0.0		0.0		0.0		0.0	
ISS	−0.3 (−1.1, 0.4)	0.400	−0.9 (−1.8, 0.1)	0.078	−0.2 (−1.0, 0.6)	0.610	−0.2 (−0.7, 0.3)	0.423	−0.3 (−0.9, 0.2)	0.217
Charlson	−1.5 (−3.8, 0.8)	0.210	−3.6 (−6.5, −0.8)	0.011	−1.1 (−3.5, 1.2)	0.353	−5.1 (−6.5, −3.7)	<0.001	−2.4 (−4.0, −0.9)	0.002
During COVID-19	−1.9 (−8.9, 5.1)	0.596	−9.8 (−18.3, −1.4)	0.022	−5.1 (−12.1, 2.0)	0.161	3.2 (−0.9, 7.4)	0.126	0.2 (−4.4, 4.7)	0.941
	**Social Functioning**		**Role-Emotional**		**Emotional Well Being**		**PCS**		**MCS**	
	**Estimate (95% CI)**	***p*** **-Value**	**Estimate (95% CI)**	***p*** **-Value**	**Estimate (95% CI)**	***p*** **-Value**	**Estimate (95% CI)**	***p*** **-Value**	**Estimate (95% CI)**	***p*** **-Value**
Age										
50–64 (ref)	0.0		0.0		0.0		0.0		0.0	
65–79	−3.1 (−10.7, 4.5)	0.420	−6.0 (−15.0, 3.0)	0.190	2.2 (−2.2, 6.7)	0.340	−1.7 (−4.5, 1.2)	0.243	−0.1 (−2.7, 2.5)	0.947
80+	0.7 (−9.3, 10.7)	0.886	2.5 (−9.4, 14.5)	0.675	5.4 (−0.4, 11.2)	0.117	1.7 (−2.0, 5.5)	0.366	0.7 (−2.8, 4.1)	0.704
Female	−8.0 (−15.2, −0.9)	0.027	−8.3 (−16.8, 0.2)	0.055	−5.0 (−9.2, −0.8)	0.018	−3.2 (−5.9, −0.6)	0.018	−1.8 (−4.2, 0.6)	0.147
Race										
Black	−3.7 (−15.7, 8.3)	0.546	−6.7 (−21.0, 7.7)	0.360	−2.8 (−9.8, 4.2)	0.429	−1.0 (−5.6, 3.5)	0.649	−2.2 (−6.3, 1.9)	0.288
Other	9.2 (−23.2, 41.5)	0.577	11.0 (−27.5, 49.6)	0.574	−0.3 (−19.1, 18.5)	0.974	7.6 (−4.6, 19.7)	0.220	−0.9 (−11.9, 10.1)	0.873
White (ref)	0.0		0.0		0.0		0.0		0.0	
Married	−1.1 (−7.9, 5.7)	0.751	−1.7 (−9.8, 6.4)	0.674	0.5 (−3.4, 4.5)	0.795	0.6 (−2.0, 3.1)	0.650	−0.6 (−3.0, 1.7)	0.595
Prior anxiety, depression										
None (ref)	0.0		0.0		0.0		0.0		0.0	
Depression only	−1.6 (−10.7, 7.5)	0.730	−0.6 (−11.4, 10.3)	0.921	−2.9 (−8.3, 2.4)	0.277	−0.3 (−3.7, 3.1)	0.875	−2.4 (−5.5, 0.7)	0.127
Anxiety Only	5.2 (−10.1, 20.6)	0.503	18.3 (−0.1, 36.6)	0.051	−6.9 (−15.9, 2.0)	0.130	3.3 (−2.4, 9.1)	0.259	−0.7 (−6.0, 4.6)	0.794
Both	−10.0 (−20.0, −0.1)	0.049	−20.5 (−32.4, −8.6)	0.001	−16.6 (−22.5, −10.8)	<0.001	1.1 (−2.7, 4.9)	0.570	−9.6 (−13.0, −6.2)	<0.001
Injury characteristics										
Fall	0.4 (−10.2, 11.1)	0.934	−5.0 (−17.8, 7.7)	0.437	−2.5 (−8.8, 3.7)	0.422	−2.6 (−6.6, 1.4)	0.203	−0.9 (−4.5, 2.8)	0.644
Motor vehicle accident	−4.5 (−15.6, 6.6)	0.429	−11.0 (−24.3, 2.2)	0.102	−1.6 (−8.0, 4.9)	0.631	−4.3 (−8.4, −0.1)	0.044	−1.4 (−5.2, 2.4)	0.458
Other (ref)	0.0		0.0		0.0		0.0		0.0	
ISS	−0.6 (−1.3, 0.2)	0.127	−0.5 (−1.4, 0.4)	0.292	−0.7 (−1.2, −0.3)	0.002	−0.2 (−0.4, 0.1)	0.288	−0.3 (−0.5, −0.02)	0.038
Charlson	−2.4 (−4.7, −0.2)	0.032	−3.6 (−6.2, −0.9)	0.009	−2.3 (−3.5, −1.0)	0.001	−1.0 (−1.8, −0.1)	0.025	−1.3 (−2.0, −0.5)	<0.001
During COVID-19	−3.7 (−10.4, 2.9)	0.272	6.6 (−1.3, 14.6)	0.103	−2.0 (−5.8, 1.9)	0.321	−1.8 (−4.3, 0.7)	0.169	0.6 (−1.6, 2.9)	0.583

Notes. SF-36 = Short-Form 36 Health survey; PCS = Physical Component Summary; MCS = Mental Component Summary; ISS = Injury Severity Score.

**Table 4:** Logistic regression results for depression/anxiety, adjusting for demographic and clinical characteristics (n = 403).

	Mild to Severe Depression		Mild to Severe Anxiety	
	OR (95% CI)	*p*-Value	OR (95% CI)	*p*-Value
Age				
50–64 (ref)	1.0		1.0	
65–79	0.72 (0.44, 1.18)	0.188	0.83 (0.50, 1.39)	0.481
80+	0.88 (0.46, 1.69)	0.708	0.55 (0.27, 1.11)	0.093
Female	1.09 (0.69, 1.72)	0.726	1.79 (1.10, 2.92)	0.019
Race				
Black	1.05 (0.49, 2.26)	0.905	1.59 (0.72, 3.55)	0.255
Other	0.27 (0.02, 3.06)	0.289	1.52 (0.19, 12.04)	0.692
White (ref)	1.0		1.0	
Married	0.99 (0.64, 1.55)	0.979	2.21 (1.37, 3.56)	0.001
Prior Anxiety, Depression				
None (ref)	1.0		1.0	
Depression only	2.54 (1.40, 4.62)	0.002	1.57 (0.86, 2.88)	0.142
Anxiety Only	2.25 (0.84, 6.01)	0.108	1.68 (0.62, 4.54)	0.308
Both	2.77 (1.42, 5.39)	0.002	4.49 (2.23, 9.04)	<0.001
Injury Characteristics				
Fall	1.15 (0.57, 2.30)	0.720	1.11 (0.53, 2.31)	0.791
Motor Vehicle Accident	1.10 (0.53, 2.26)	0.803	1.30 (0.61, 2.78)	0.495
Other (ref)	1.0		1.0	
ISS	1.05 (0.99, 1.10)	0.077	1.07 (1.02, 1.13)	0.012
Charlson	1.26 (1.07, 1.47)	0.005	1.23 (1.05, 1.43)	0.010
During COVID-19	0.79 (0.51, 1.22)	0.296	1.05 (0.67, 1.65)	0.835

## Data Availability

The original contributions presented in this study are included in the article/Supplementary Materials. Further inquiries can be directed to the corresponding author.

## Supplementary Materials

The following supporting information can be downloaded at https://www.mdpi.com/article/10.3390/geriatrics11040090/s1, Limited Study Data Set.

## Abbreviations

The following abbreviations are used in this manuscript:

COVID-19	Coronavirus Disease 2019
TMH	Trauma Medical Home
ISS	Injury Severity Score
CDC	Centers for Disease Control and Prevention
PHQ-9	Patient Health Questionnaire -9
GAD-7	Generalized Anxiety Disorder-7
SF-36	Short-Form 36 Health Survey
PCS	Physical Component Summary
MCS	Mental Component Summary
EMR	Electronic Medical Record
ICD-9	International Classification of Diseases, Ninth Revision
ICD-10	International Classification of Diseases, Tenth Revision
OR	Odds Ratio
CI	Confidence Interval
SD	Standard Deviation
PTSD	Post-Traumatic Stress Disorder
COADS	Caregiver Outcomes of Alzheimer’s Disease Screening
UK	United Kingdom
AI	Artificial Intelligence
